# Twenty years' follow-up of a de-epithelialised turnover flap

**DOI:** 10.4103/0970-0358.44944

**Published:** 2008

**Authors:** A. Gopalakrishna, T. V. Pavan Kumar

**Affiliations:** Department of Plastic Surgery, Deccan College of Medical Sciences and Allied Hospitals, Hyderabad, India

**Keywords:** De-epithelialised turnover flap, long-term follow-up, Thatte's flap, tendo-achilles

## Abstract

The authors have used the de-epithelialised turnover flap (Thatte's Flap) for covering compound defects over the tendo-achilles in 31 patients in the period covering 1980–2000. Two of these patients have come up for follow-up after 20 years. In this late follow-up, the results are good and the tendon which was bridged by the dermis of the flap is functioning to allow the patients to stand on their toes. The de-epithelialised turnover flap is a simple and easy technique giving good long-term results. In spite of all the new advances in flap coverage, the de-epithelialised turnover flap is a good alternative for the tendo-achilles area in the armamentarium of a plastic surgeon.

The de-epithelialised turnover flap was first described in 1981 by Thatte.[1] It was a simple concept to cover defects in any part of the body without the need for any sophisticated equipment. In the ‘80s, this flap was extensively used[2–7] to cover defects which were difficult to treat with flaps available at the time, with the exception of free flaps, which again, were not as commonly done in India at that time.

With the advent of distally based flaps in the leg and with more extensive use of microsurgery, the de-epithelialised flap has been used less frequently.

In India, long-term follow-up is a problem because of poor compliance from the patients. There are many reasons for this: the cost of travelling and loss of wages prevents the patients from attending follow-up unless it is absolutely essential.

At the beginning of his practice in the early 80's, working alone and without training in microsurgery, the first author found this flap extremely useful for the management of a compound injury of the tendo-achilles.

## PROCEDURE

**Figure 1:**
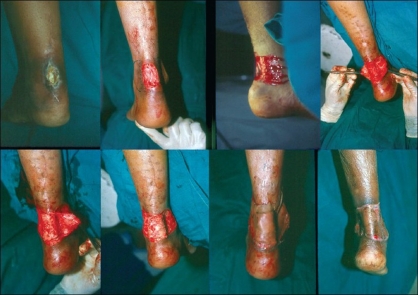
Surgical procedure

The defect on the tendo-achilles was marked as were two flaps, one on either side. The whole area was de-epithelialised and flaps were raised. The necrotic portion of the tendo-achilles was excised. One of the de-epithelialised flaps was sutured to the tendon, forming a bridge for replacing the partially or completely lost tendon. The second flap was used as cover and the exposed undersurface of the flap and the two secondary defects were covered with a single sheet of a split skin graft taken from the thigh. Dressing and POP were applied; dressings were changed from the 5^th^ to 7^th^ day [Figure 1].

Between 1980 and 2000, the first author performed this procedure on 31 patients. However, as is usual in Indian conditions, the patients were lost to follow-up after six months to a year. Recently, two of these patients returned for follow-up.

Patient 1: A former football player aged 28 years at the time of injury in 1984 came with a history of injury to the tendo-achilles, which was repaired primarily elsewhere. There was necrosis of the overlying skin and complete necrosis of the tendo-achilles. In 1984, he underwent surgery where bilateral turnover flaps were done as described above. The patient attended follow-up for about six months for minor complaints of occasional cyst formation under the graft.

The patient came for follow-up in 2004 with a history of ulcer at the operated site for two months. There was a small posttraumatic ulcer caused by faulty footwear which was rubbing against the less sensitive skin-grafted area. The ulcer healed on changing footwear and dressings over about a week to ten days.

Patient 2: A 50 year-old farmer was seen in 1986 with a history of injury to the tendo-achilles, which was repaired primarily resulting in a loss of skin and partial necrosis of the tendo-achilles. He underwent the same procedure as described above. He attended follow-up for about 6–8 months. This patient was seen in 2008 when he came as a companion with another patient. He did not have any problems while walking.

**Figure 2:**
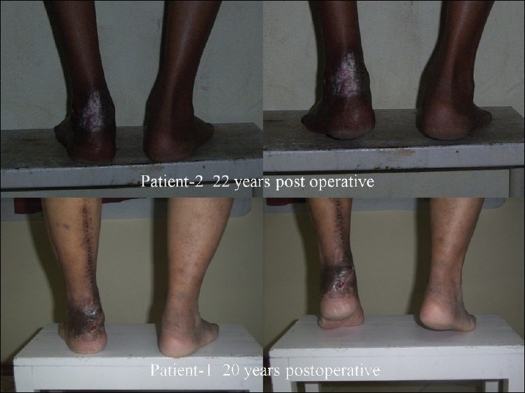
Long-term postoperative result

Both patients could stand on their toes. They were not taking part in strenuous activities like running, but had no problem in normal walking [Figure 2].

Considering the rarity of long-term, postoperative follow-up in Indian patients, the authors would like to present these two cases which illustrate that the de-epithelialised flap, although not in much use today, still deserves a place in the armamentarium of a plastic surgeon for its simplicity, ease of execution, and good results, at least for the above indication.

## DISCUSSION

The de-epithelialised turnover flap (Thatte's flap) is a simple, easy-to-perform procedure giving consistently good results. It has its drawbacks in the form of cyst formation, breakdown of graft, and poor appearance. The advent of better flaps with better vascularity and aesthetic appearance has resulted in this flap being abandoned in favour of better alternatives. However, the authors feel that in the specific indication of compound injury to the tendo-achilles, Thatte's flap has a role to play. The flap can be used for cover as well as for bridging the gap in the tendon.

Long-term follow-up is rare in India. The cost of travelling and loss of wages are probably the main reasons why our patients do not attend follow-up, unless they have a problem. Out of 31 patients on whom this procedure was performed, two have returned for a follow-up after 20 years.

In this long-term follow-up, we find that both patients are mobile, symptom-free, and performing their normal daily routine comfortably. Both patients are able to stand on their toes, indicating that the repaired tendo-achilles is functioning. Of course, both are sedentary and do not take part in running or similar activities that impose any kind of strain on the tendon.

## CONCLUSION

The de-epithelialised turnover flap is a useful procedure for the repair of compound injury to the tendo-achilles that gives good long-term results. It is a good flap to have in one's armamentarium, in spite of all the new advancements in flap surgery.
